# Un cas d'hamartome musculaire cervical retrouvé à Antananarivo

**DOI:** 10.11604/pamj.2015.22.283.8284

**Published:** 2015-11-23

**Authors:** Rex Mario Razafindrakoto, Odilon Laza

**Affiliations:** 1 Service d'Oto-rhino-laryngologie et de Chirurgie Cervico-faciale, Centre Hospitalier Universitaire d'Andohatapenaka, Antananarivo, Madagascar; 2 Département d'Anatomie Pathologique, Centre Hospitalier Universitaire Joseph Ravoahangy Andrianavalona, Antananarivo, Madagascar

**Keywords:** Hamartome musculaire cervical, tuméfaction latéro-cervicale, cervicotomie, cervical muscle hematoma, latero-cervical swelling, cervicotomy

## Image en medicine

Une fillette de dix ans nous a été amenée en consultation pour une tuméfaction latéro-cervicale gauche apparue deux mois auparavant. Cette masse n'a occasionné ni fièvre ni douleur, mais son augmentation progressive de volume a inquiété les parents, motivant la consultation. On a retrouvé à l'examen clinique une formation ovalaire à grand axe vertical, de consistance ferme, mobile par rapport aux plans superficiels et profonds, indolore à la palpation, non battante, non soufflante, n'ascensionnant pas à la déglutition et recouverte d'une peau d'apparence normale (A). Une échographie cervicale a révélé une masse tissulaire, hypoéchogène, bien limitée, située au contact du lobe gauche de la glande thyroïde, mesurant 52,1 millimètres de hauteur sur 27,6 millimètres de largeur (B). Une cervicotomie menée sous anesthésie générale a permis l'ablation d'une masse vascularisée en surface (C,D), enlevée en totalité, sans effraction capsulaire (E). L'examen anatomopathologique de la pièce opératoire a retrouvé une prolifération de fibres musculaires lisses et a conclus au diagnostic histologique d'hamartome musculaire (F). Un hamartome est une dysembryoplasie liée à une malformation tissulaire d'aspect tumoral, composée d'un mélange anormal d’éléments constitutifs normalement présents dans l'organe où ils se sont développés. L'ablation chirurgicale peut être proposée pour des hamartomes qui ont entrainé une gêne fonctionnelle ou une gêne esthétique, comme chez notre patiente. Aucune récidive n'a été notée aux contrôles effectués un et six mois après la cure chirurgicale.

**Figure 1:**
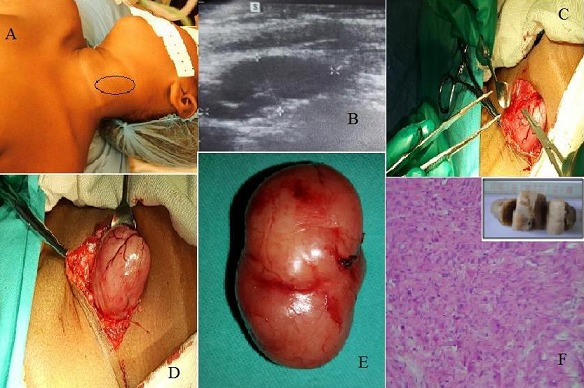
A): installation chirurgicale de la patiente; B): echographie cervicale montrant une masse tissulaire, hypoéchogène, bien limitée, mesurant 52,1 x 27,6 millimètres; C): dissection per-opératoire de l'hamartome musculaire cervical (1); D): dissection per-opératoire de l'hamartome musculaire cervical (2); E): pièce opératoire; F): découpe de la pièce opératoire au microtome et lame histologique montrant une prolifération de fibres musculaires lisses (grossissement x 100)

